# Targeted sequencing of established and candidate colorectal cancer genes in the Colon Cancer Family Registry Cohort

**DOI:** 10.18632/oncotarget.18596

**Published:** 2017-06-21

**Authors:** Leon Raskin, Yan Guo, Liping Du, Mark Clendenning, Christophe Rosty, Colon Cancer Family Registry (CCFR), Noralane M. Lindor, Stephen B. Gruber, Daniel D. Buchanan

**Affiliations:** ^1^ Division of Epidemiology, School of Medicine, Vanderbilt University Medical Center and Vanderbilt Ingram Comprehensive Cancer Center, Nashville, TN, USA; ^2^ Center for Quantitative Sciences, Vanderbilt University Medical Center and Vanderbilt Ingram Comprehensive Cancer Center, Nashville, TN, USA; ^3^ Colorectal Oncogenomics Group, Genetic Epidemiology Laboratory, Department of Pathology, University of Melbourne, Parkville, Victoria, Australia; ^4^ Envoi Specialist Pathologists, Herston, Queensland, Australia; ^5^ University of Queensland, School of Medicine, Herston, Queensland, Australia; ^6^ Department of Health Sciences Research, Mayo Clinic, Scottsdale, AZ, USA; ^7^ USC Norris Comprehensive Cancer Center, Los Angeles, CA, USA; ^8^ Department of Preventive Medicine, University of Southern California, Los Angeles, CA, USA; ^9^ Genetic Medicine and Familial Cancer Centre, The Royal Melbourne Hospital, Parkville, Victoria, Australia

**Keywords:** targeted sequencing, DNA pooling, rare variants, hereditary colorectal cancer, Colon Cancer Family Registry

## Abstract

The underlying genetic cause of colorectal cancer (CRC) can be identified for 5-10% of all cases, while at least 20% of CRC cases are thought to be due to inherited genetic factors. Screening for highly penetrant mutations in genes associated with Mendelian cancer syndromes using next-generation sequencing (NGS) can be prohibitively expensive for studies requiring large samples sizes. The aim of the study was to identify rare single nucleotide variants and small indels in 40 established or candidate CRC susceptibility genes in 1,046 familial CRC cases (including both MSS and MSI-H tumor subtypes) and 1,006 unrelated controls from the Colon Cancer Family Registry Cohort using a robust and cost-effective DNA pooling NGS strategy. We identified 264 variants in 38 genes that were observed only in cases, comprising either very rare (minor allele frequency <0.001) or not previously reported (n=90, 34%) in reference databases, including six stop-gain, three frameshift, and 255 non-synonymous variants predicted to be damaging. We found novel germline mutations in established CRC genes *MLH1*, *APC*, and *POLE,* and likely pathogenic variants in cancer susceptibility genes *BAP1, CDH1, CHEK2, ENG,* and *MSH3*. For the candidate CRC genes, we identified likely pathogenic variants in the helicase domain of *POLQ* and in the *LRIG1*, *SH2B3*, and *NOS1* genes and present their clinicopathological characteristics. Using a DNA pooling NGS strategy, we identified novel germline mutations in established CRC susceptibility genes in familial CRC cases. Further studies are required to support the role of *POLQ*, *LRIG1*, *SH2B3* and *NOS1* as CRC susceptibility genes.

## INTRODUCTION

The underlying genetic cause of colorectal cancer (CRC) can only be identified for 5-10% of cases despite approximately 20% of all CRC cases thought to be due to inherited genetic factors [1], highlighting that the genetic cause for the majority of the heritable CRC is still unknown [2]. Germline mutations in the DNA mismatch repair (MMR) genes [3] and the *APC* [4] gene were discovered over 20 years ago, accounting for 2-5% of CRC overall. Since then, linkage studies have led to some progress in identifying additional highly penetrant genes including *MUTYH* [5], *STK11* [6], *BMPR1A* [7], *SMAD4,* and *PTEN* [8], when combined might explain a further 1% of CRC. Genome-wide association-based studies (GWAS) have identified common germline alleles, but all have been weakly associated with CRC risk and collectively are likely to explain only a few percent of the missing heritability for CRC [9]. Therefore, while a number of established hereditary CRC genes exist, the cause of the majority of inherited CRC remains explained.

Up to half of the CRC cases with a very strong family history of CRC (fulfilling the Amsterdam criteria I) have microsatellite stable (MSS) tumours and do not carry an inherited MMR gene mutation [10]. For almost all of these families, no mutation can be identified which has important negative clinical implications for family members. These families have been named “Familial Colorectal Cancer Type X” (FCCTX) [11]. FCCTX is probably not a single disorder, rather, it is more likely to be a heterogeneous group of CRCs including: CRC cases with a chance aggregation of CRC in their relatives (lifetime risk of CRC is 5% in the general population); CRC cases with an undiagnosed syndrome e.g. undetected Lynch syndrome or *MUTYH*-associated polyposis [12]; but for the majority of CRC cases it will be yet-to-be-discovered genetic mutations.

Next-generation sequencing (NGS) (whole genome or exome) has facilitated further discovery of cancer susceptibility genes including *RECQL*, *FANCM*, *FANCC*, *XRCC2*, *POT1*, and *BAP1* for breast and melanoma [13], and *POLE*, *POLD1*, and *NTHL1* for CRC [14, 15]. Highly penetrant cancer mutations, such as those observed in the *APC* or MMR gene mutations in CRC and *BRCA1/2* genes in breast cancer, are rare and usually population-specific. Therefore, screening for mutations in these and other cancer susceptibility genes using NGS requires large sample sizes, which makes this strategy prohibitively expensive.

The cost of whole genome sequencing has dropped from about $10 million in 2007 to a reasonable $3,000-$4,000 per genome nowadays [16]. However, it is widely agreed that the cost of variant interpretation is not going down anytime soon. While sequencing projects, such as NHLBI GO Exome Sequencing Project (ESP) and 1000 Genomes Project, have generated an enormous amount of data on common and rare variants, the interpretation of the significance of these variants in the etiology of hereditary syndromes is limited. Genome or exome analyses of well-defined case-control studies to identify rare, highly penetrant mutations associated with hereditary syndromes are still prohibitively expensive and frequently rely on the sequencing of cases only to reduce the cost.

Common variants and very rare variants have not explained heritability of complex diseases and the research paradigm has shifted towards the role of large sets of rare variants with moderate effect sizes [17, 18]. While very rare variants do not explain the entirety of missing heritability of complex diseases, they may help to elucidate new mechanisms of the development of a disease. Moreover, rare, highly penetrant mutations have great importance for genetic counseling, disease screening, and primary prevention of hereditary cancer. Recent identification of *POLE* and *POLD1* genes with mutations predisposing to hereditary CRC [15] are the perfect example of rare, highly penetrant genes that have expanded our understanding of CRC pathogenesis by implicating inherited impairment of DNA base-excision repair in CRC predisposition.

The aim of the study was to apply a DNA pooling NGS strategy to screen 40 established or candidate CRC susceptibility genes in order to identify rare, likely pathogenic variants across a study of 1,046 familial CRC cases and 1,006 controls from the Colon Cancer Family Registry (CCFR). The DNA pooling strategy presented here is at least five times less expensive than traditional NGS approaches and could be applied to other familial diseases.

## RESULTS

### Analysis of very rare variants

**Figure 1:**
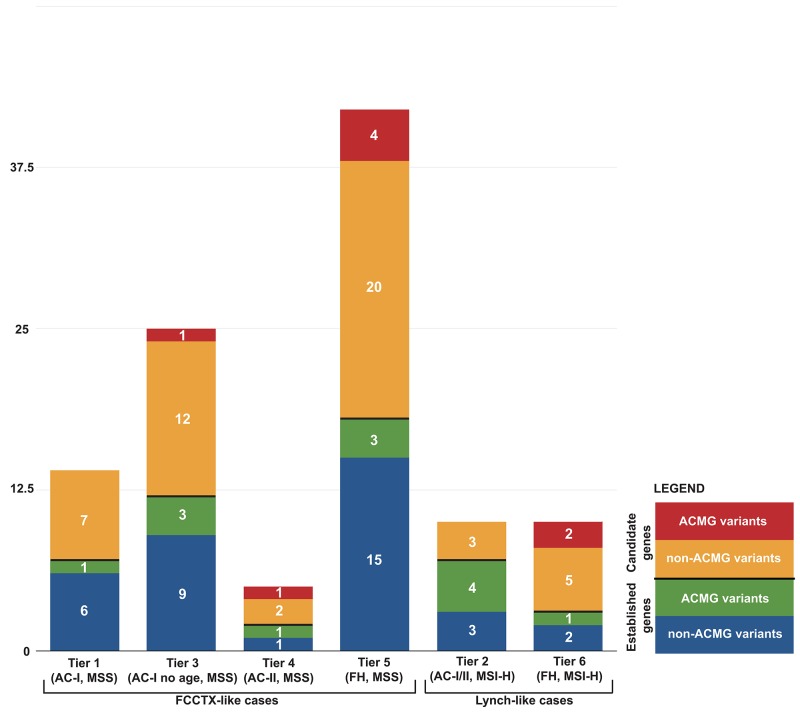
Distribution of 106 germline variants from 40 established and candidate CRC genes by case tiers

The characteristics of the familial CRC cases according to their recruitment category (Tiers 1 to 6) and controls are shown in Table 1 where 89% of the cases were whites. A total of 9,985 unique non-synonymous, stop-gain, stop-loss, and frameshift variants in 40 genes were identified across all cases and controls. Subsequent variant filtering based on allele frequency and predicted functional impact identified a total of 264 rare, likely deleterious variants in 38 genes (no variants met selection criteria in *PTEN* or *STK11*) in 287 out of 1046 CRC cases, not found in the tested controls and observed at a very low frequency or absent in reference population datasets (MAF <0.001). Among all variants, six were stop-gain, three frameshifts, and 255 were non-synonymous variants. Out of 264 rare variants, 20 were found in more than one pool (Supplementary Table 1). The frequency of likely deleterious variants identified across the six Tiers ranged from 8.2% (Tier 5) to 15.6% (Tier 2) (Figure 1). A total of 24 MMR variants were identified across all cases, including those with MSS CRCs, with Tier 2 cases having the highest proportion of MMR gene variants identified. A total of 6 *POLE* and 2 *POLD1* likely deleterious variants were identified across all CRC cases tested, all of which had tumors that were MSS. Considering only those variants that met the more stringent ACMG criteria of pathogenicity [19], reduced the number of variants from 264 to 21 variants for both established and candidate CRC genes, the majority of which were in Tier 2 cases (4/21) (Table 2).

**Table 1:** Characteristics of the study sample

	FCCTX-like cases				Lynch-like cases		Cases, n (%)	Controls, n (%)	Total
Tier	Tier 1	Tier 3	Tier 4	Tier 5	Tier 2	Tier 6			
Tier criteria	AC-I	AC-I (No age)^*^	AC-II	CRC<60 ≥1 FDR/SDR	AC-I/II	CRC ≥1 FDR/SDR			
Tier MSI status	MSS	MSS	MSS	MSS	MSI-H	MSI-H			
**Total**	139 (13.3%)	202 (19.3%)	36 (3.4%)	501 (47.9%)	64 (6.1%)	104 (10%)	1046	1006	**2052**
**Sample origin**									
Fred Hutchinson Cancer Research Center	6	22	5	67	6	15	121 (12%)	504 (50%)	**625**
University of Hawaii	5	13	-	36	-	6	60 (5%)	110 (11%)	**170**
Cancer Care Ontario	26	84	10	96	13	27	256 (25%)	26 (3%)	**282**
USC Consortium	15	32	10	52	25	17	151 (14%)	0	**151**
University of Melbourne	58	34	5	155	16	20	288 (28%)	256 (25%)	**544**
Mayo Clinic	29	17	6	95	4	19	170 (16%)	110 (11%)	**280**
**Age** (mean (range))	55 (26–87)	63 (23-92)	58 (39-76)	53 (29-94)	55 (20-87)	58 (29-84)	56 (20-94)	61 (43-84)	
**Sex**									
Male	61	105	15	251	31	49	512 (49%)	395 (39%)	**907**
Female	78	97	21	249	33	55	533 (51%)	611 (61%)	**1144**
Unknown	0	0	0	1	0	0	1 (0%)	0	**1**
**Race**									
White	127	180	36	436	59	96	934 (89%)	863 (85%)	**1797**
Black	1	1	0	9	0	0	11 (1%)	20 (2%)	**31**
Asian	6	17	0	45	2	7	77 (7%)	97 (10%)	**174**
American Indian	1	1	0	2	0	0	4 (1%)	2 (1%)	**6**
Other or unknown	4	3	0	9	3	1	20 (2%)	24 (2%)	**44**
**CRC site**									
Large intestine	102	140	23	374	59	98	796 (76%)	-	**796**
Rectum	37	57	13	123	5	6	241 (23%)	-	**241**
Appendix	0	4	0	4	0	0	8 (1%)	-	**8**
**MSI**									
MSS	112	174	33	417	-	-	736 (70%)	-	**736**
MSI-Low	27	28	3	84	-	-	142 (14%)	-	**142**
MSI-High	-	-	-	-	64	104	168 (16%)	-	**168**

AC – Amsterdam criteria, MSI – microsatellite instability, MSS – microsatellite stable, MSI-H – highly microsatellite unstable, FDR – first degree relative, SDR – second degree relative.

^*^AC-I (no age) describes Tier 3 cases that fulfill all AC-I criteria except “At least 1 of the cancers diagnosed before age 50”.

**Table 2:** Distribution of 99 identified germline variants from 40 CRC genes by case tiers

		FCCTX-like cases				Lynch-like cases	
Tier		Tier 1	Tier 3	Tier 4	Tier 5	Tier 2	Tier 6
Criteria		AC-I	AC-I (no age)^**^	AC-II	CRC<60 ≥1 FDR/SDR	AC-I/II	CRC ≥1 FDR/SDR
MSI status		MSS	MSS	MSS	MSS	MSI-H	MSI-H
Established CRC genes	All variants	7 (5.0%)	12 (5.9%)	2 (5.6%)	18 (3.6%)	7 (10.9%)	3 (2.9%)
	ACMG pathogenic variants	1 (0.7%)	3 (1.5%)	1 (2.8%)	3 (0.6%)	4 (6.3%)	1 (1.0%)
	Genes with variants	APC, MLH1, MSH2^*^, POLE, TGFBR2	MLH3^*^, MSH2, MSH6^*^, POLD1, POLE	MUTYH, POLE^*^	APC^*^, AXIN2, MLH1, MLH3, MSH2^*^, MSH6, MUTYH, POLD1, POLE^*^	MLH1^*^, MSH2^*^, MSH6^*^	MLH1, MSH2, MSH6^*^
Candidate CRC genes	All variants	7 (5.0%)	13 (6.4%)	3 (8.3%)	24 (4.8%)	3 (4.7%)	7 (6.7%)
	ACMG pathogenic variants	-	1 (0.5%)	1 (2.8%)	4 (0.8%)	-	2 (1.9%)
	Genes with variants	*ALPK2, CDH1, LAMA2, MSH3, NOS1, PREX1*	ALPK2, BLM, LAMA2, MSH3, NOS1, PALB2, POLQ, PTCH1, SH2B3^*^	LAMA2, MSH3, SH2B3^*^	ALPK2, BAP1, BLM, CDH1, HELQ, LAMA2, LRIG1, MSH3, NOS1^*^, POLQ^*^, PREX1, PTCH1, SH2B3^*^	*HELQ, PALB2, PTCH1*	CHEK2^*^, ENG, LAMA2, LRIG1, MSH3, NOS1, POLQ^*^
**All ACMG pathogenic variants**		**1/139 (0.7%)**	**4/202 (2.0%)**	**2/36 (5.6%)**	**7/501 (1.4%)**	**4/64 (6.3%)**	**3/104 (2.9%)**
**All variants**		**14/139 (10.0%)**	**25/202 (12.4%)**	**5/36 (13.9%)**	**42/501 (8.4%)**	**10/64 (15.6%)**	**10/104 (9.6%)**

^*^Genes with ACMG classified mutations.

^**^AC-I (no age) describes Tier 3 cases that fulfill all AC-I criteria except “At least 1 of the cancers diagnosed before age 50”.

AC – Amsterdam criteria, MSI – microsatellite instability, MSS – microsatellite stable, MSI-H – highly microsatellite unstable, FDR – first degree relative, SDR – second degree relative, ACMG – American College of Medical Genetics.

A subset of 264 rare variants were selected for Sanger sequencing validation of those variants predicted to be deleterious by either SIFT, PolyPhen2, or MutationTaster, listed as pathogenic in ClinVar, or were stop-gain variants (n=108) in 348 cases from 116 pools. We found no variants in *STK11*, *PTEN*, *GREM1*, and *WDR78*; and we found only variants predicted to be benign in *EPCAM*, *TP53*, *BRAP*, *ENTPD7*, *MET*, and *FZD7*. Six variants failed in Sanger sequencing for various reasons including inability to design primers in repeat-prone loci, mispriming, and technical error. Two variants were not found on chromatograms, although high depth of targeted sequencing of the locus (84/295 and 196/548 reads) suggests a technical error of using wrong DNA sample for Sanger sequencing. Unfortunately, we did not have enough DNA to repeat sequencing. Considering these two variants as “unconfirmed”, we had sensitivity of >98% (99/101 variants). Thus, Sanger sequencing was successful for 99 variants from 106 pools (Table 3 and Supplementary Table 2). Eight variants were found in two cases and one variant was found in three cases. Out of 99 variants 24 (24%) were novel.

**Table 3:** The 40 established and candidate CRC susceptibility genes used for targeted sequencing and the distribution of 99 identified variants in 106 cases

		Number of patients with identified variants						
		FCCTX-like cases				Lynch-like cases		Total
		Tier 1	Tier 3	Tier 4	Tier 5	Tier 2	Tier 6	
Criteria		AC-I	AC-I (No age)^****^	AC-II	CRC<60 ≥1 FDR/SDR	AC-I/II	CRC ≥1 FDR/SDR	
MSI status		MSS	MSS	MSS	MSS	MSI-H	MSI-H	
**Established CRC genes**	*BMPR1A, EPCAM, PMS2, PTEN, SMAD4, STK11, TP53*	-	-	-	-	-	-	**0**
	*AXIN2*	-	-	-	1	-	-	**1**
	*TGFBR2*	1	-	-	-	-	-	**1**
	*POLD1*	-	1	-	1	-	-	**2**
	*MUTYH*	-	-	1	2	-	-	**3**
	*APC*	2	-	-	2^*^	-	-	**4**
	*MLH1*	1	-	-	1	2	1	**5**
	*POLE*	1	1	1	3	-	-	**6**
	*MLH3*	-	4	-	3	-	-	**7**
	MSH6^**^	-	2^*^	-	2	2^*^	1^*^	**7**
	*MSH2*	2	4	-	3	3	1	**13**
**Candidate CRC genes**	*BRAP, CTNNB1, ENTPD7, GREM1, MET, FZD7, WDR78*	-	-	-	-	-	-	**0**
	*BAP1*	-	-	-	1	-	-	**1**
	*CHEK2*	-	-	-	-	-	1^*^	**1**
	*ENG*	-	-	-	-	-	1	**1**
	*BLM*	-	1	-	1	-	-	**2**
	*PREX1*	1	-	-	1	-	-	**2**
	*HELQ*	-	-		1	1	-	**2**
	*PALB2*	-	1	-	-	1	-	**2**
	POLQ^**^	-	1	-	1	-	1	**3**
	*CDH1*	1	-	-	2	-	-	**3**
	*LRIG1*	-	-	-	3	-	1	**4**
	*PTCH1*	-	1	-	2	1	-	**4**
	*ALPK2*	1	2	-	2	-	-	**5**
	*MSH3*	1	2	1	1	-	1	**6**
	*LAMA2*	1	2	1^*^	1^*^	-	1	**6**
	SH2B3^***^	-	1	1	5	-	-	**7**
	NOS1^**^	2	2	-	3	-	1	**8**
**Total**		**14**	**25**	**5**	**42**	**10**	**10**	**106**

^*^Including one nonsense variant, all other variants are missense.

^**^One variant was found in two patients.

^***^One variant was found in two patients.

^****^AC-I (no age) describes Tier 3 cases that fulfill all AC-I criteria except “At least 1 of the cancers diagnosed before age 50”.

### Variants within the MMR genes

While 34% (90/264) of the rare variants have never been reported before, almost all (33/36) MMR genes (*MLH1*, *MSH2*, *MSH6*, and *PMS2*) variants have been reported in dbSNP (Supplementary Table 1). However, only 8 out of 36 MMR variants had annotation in ClinVar database (6 pathogenic and 2 benign), other variants did not have sufficient evidence to determine their effect and were classified as variants of uncertain clinical significance (VUS) (Supplementary Table 1).

We found 10 MMR gene variants (3 *MLH1*, 4 *MSH2*, 3 *MSH6*) in Lynch-like cases (Tiers 2 and 6) and 14 MMR gene variants (2 *MLH1*, 8 *MSH2*, 4 *MSH6*) in FCCTX-like cases (Tiers 1, 3, 4, 5), which correspond to 6% and 1.6% of all cases respectively. Two variants in Lynch-like cases were nonsense, while all variants in FCCTX-like cases were missense. Median age at diagnosis for MMR variant carriers was different between Lynch-like (50.5 years, range 28 – 62) and FCCTX-like cases (57 years, range 36 – 73), although not statistically significant (p=0.144). In Lynch-like cases 50% (5/10) of the variants were pathogenic according to ACMG criteria in comparison to 21% (3/14) of variants in FCCTX-like cases. In 67% of Lynch-like cases MMR proteins had impaired expression in IHC, while all FCCTX-like cases had intact MMR protein expression.

Among five ***MLH1*** variants, we found two known mutations, putatively pathogenic variants p.R100P, p.R226L and a novel p.A125E in three AC-I positive patients with MSI-High CRC diagnosed before 50 years (Tier 2). We found 12 ***MSH2*** variants in 13 patients; 70% of the patients had multiple cancers (Supplementary Figure 1). The *MSH2* p.G692V variant, currently considered a VUS in ClinVar, was identified in a patient with metachronous CRC at 29 and 44 years demonstrating MSI-H and loss of MSH2/MSH6 protein expression, providing further support for variant pathogenicity. Six ***MSH6*** variants were found in seven patients. *MSH6* p.K295I, p.S541R, and p.T767S were found in cases with CRC diagnosed before 50 years of age. The *MSH2* p.H46Q classified as VUS by ClinVar was found in two cases, both with normal protein expression of MSH2, and we identified two cases carrying the nonsense *MSH6* mutation p.R911^*^ and a further case carrying the p.R298^*^ mutation. Some variants classified as VUS were found in patients with young age metachronous CRC and/or endometrial cancer (*MLH1* p.A125E, *MSH2* p.H466R, and *MSH6* p.T767S). Of note, over 20% (5/24) of sequenced MMR variants were identified in East Asians and Native American, although 89% of the all cases from this study were white.

### Other established CRC genes

We found a novel nonsense ***APC*** p.C1410^*^ variant in male with metachronous MSS CRC at 28, 48, and 49 years. *APC* variants p.T1160K and p.A1358T (both VUS in ClinVar) were found in patients with MSS CRC at 43 and 51 years respectively (Supplementary Figure 2). All *APC* patients had polyps except p.T1160K carrier. Very rare (MAF<0.0003) p.R594W variant in ***AXIN2*** was found in male with MSI-Low CRC at 45 years. We found one ***BMPR1A*** variant p.R406C in two patients with MSI-High CRC below age 53 and below age 47 years old (individual genotypes were not available). Three ***MUTYH*** heterozygous variants predicted to be deleterious and highly conserved (all VUS in ClinVar) were found in patients with CRC before age 50 years but no polyps. Of the two ***POLD1*** variants identified (neither in ClinVar), p.Q411H (melanoma at 28 years and MSS CRC at 58 years) and p.Q684H (MSS CRC at 55 years), only the former resided within the exonuclease domain. In ***POLE*** exonuclease domain, we found one novel (p.D301G) and one previously reported (p.R231C) variants. Novel (p.N143D) and previously reported (p.H144R) variants were identified close to the exonuclease domain of *POLE*. All six ***MLH3*** variants (p.A1394T, p.N1147I, p.L1111F, p.D1049N, p.L880V, and p.F168S) were in cases with MSS CRC before age 60 (Supplementary Figures 3 and 4). *MLH3* p.F168S was found in two females with CRCs at 55 and 65 years from families that met AC-I without age restriction. ***TGFBR2*** p.G169R variant was found in male with MSS CRC at 31 years from AC-I positive family.

### Candidate CRC genes

We found p.R389C variant in ***BAP1,*** a well-established tumor suppressor gene, in the case with CRC, squamous cell carcinoma (SCC), and basal cell carcinoma (BCC) at age 66. ***BLM*** variants (p.S897C and p.Y1044C) were found in patients with metachronous CRCs, Japanese male (CRC at 48 and 72 years) and white female (CRC at 72 and 79 years). Three ***CDH1*** variants (p.R335Q, p.L630V, and p.A817V) listed as VUS in ClinVar were found in cases with CRC before age 60 and no history of gastric cancer. While ***CHEK2*** is considered a low-risk gene for CRC, we found previously reported truncating *CHEK2* mutation p.R95^*^ in a 52 year old woman diagnosed with MLH1/PMS2 deficient MSI-High CRC. In ***MSH3*** only p.A1064T was found in a female with MSI-High CRC at 62 years, other variants (p.D143N, p.L432W, p.I440M, p.V682L, and p.M892V) were in cases with MSS and MSI-Low CRC between 51 and 74 years. However, all *MSH3* variants were heterozygous. Two of the tumor suppressor ***PTCH1*** variants (p.R1391W and p.T1106M) were found in cases with MSS CRC before 50 years. ***ENG*** exon 12 variant p.T550M carrier had polyps at 53 years and CRC at 70 years.

Variants in ***LRIG1***, ***PREX1***, ***NOS1***, and ***SH2B3*** have been recently found to be associated with CRC in a large GWAS [20]. Variants in ***LRIG1***, a known tumor suppressor downregulated in CRC [21], were identified in cases with CRC before age 50 (adjacent p.V805I, p.R738W, and p.R723C). In ***PREX1***, another known tumor suppressor, we found two variants (p.R1243W and p.V569M) in cases with MSS CRC at 55 and 50 years. Seven ***NOS1*** variants were found in cases with CRC between 36 and 72 years (Supplementary Figures 5 and 6). All five variants in the tumor suppressor ***SH2B3*** were found in cases with MSS CRC. A highly conserved *SH2B3* p.E395K was identified in three cases with MSS CRC at 47, 54, and 62 years. Another conserved *SH2B3* variant p.N271T was found in a female with CRC and breast IDC at 43 years. Among others, *SH2B3* p.I568T was found in a case with synchronous MSS CRCs at 45 years and p.P512T was found in a case with MSS CRC at 50 years.

In ***POLQ***, a DNA polymerase involved in DNA repair with helicase activity, we found two variants predicted to be deleterious: p.P291L (C-terminal helicase domain) in two cases with CRC at 24 and 55 years and p.Y2420C (polymerase domain) in a case with CRC at 50 years. ***LAMA2*** is a methylation target in CRC [22] with mutations predisposing to congenital muscular dystrophy type 1A (MCD1A). Nonsense variants were found in a female with MSS CRC at 73 years and genital malignancy at 68 years (p.Y1334^*^) and a male with MSI-Low CRC at 53 years and prostate adenocarcinoma at 57 years (p.R2578^*^). We found novel ***PALB2*** variant p.H1076Y in a Chinese male with MSS CRC at 55 years.

## DISCUSSION

In this study, we present a DNA pooling targeted NGS analysis of CRC-related and candidate genes in a large cohort of familial CRC patients Over a third of 264 identified variants were novel. Variants classified as pathogenic by ACMG (Table 2) represent the clinically actionable mutations; however, ACMG classification is partly based on prior publications and recently established CRC genes or candidate CRC genes have insufficient functional data yet. Variants classified as VUS by ACMG criteria include candidates suggestive of being pathogenic, such as *POLE* p.H144R (MSS CRC at 48 years), *BLM* p.Y1044C (MSS CRC at 48 and 72 years), and *MLH1* p.R100P (MSI-H CRC at 28 years). All VUS were predicted to be deleterious by several bioinformatics tools and the majority are highly conserved, which suggests that these variants may include genuine CRC mutations. ACMG criterion PS4, the prevalence of the variant in affected individuals is significantly increased compared with the prevalence in controls, is particularly problematic when very rare or private mutations are studied.

Despite previous screening of the CRC-affected individuals using dHPLC, Sanger sequencing and MLPA, variants in the core MMR genes (MLH1, MSH2, and MSH6), comprised 22.6% (24/106) of the cases identified to carry one of the rare, predicted pathogenic variants identified in our study. This included carriers who had developed MSI-High CRCs (Lynch-like cases) but also those who developed MSS CRCs from within the FCCTX-like cases. MMR gene mutation carriers who develop MSS CRCs has been reported previously, particularly for MSH6 mutation carriers with missense mutations. We observed MSS CRCs in not only MSH6 missense variant carriers but also for carriers of missense variants in the MSH2 and MLH1 genes. While further validation of the pathogenicity of these MMR gene missense variants is needed, the observation from our study that six MMR gene missense variants classified as VUS by ClinVar were identified in individuals who developed MSS CRCs may warrant further consideration by organizations working towards implementing population-based screening programs for Lynch syndrome that are based on screening CRCs via MMR immunohistochemistry for evidence of tumor mismatch repair deficiency before subsequent germline MMR gene testing. Some of the identified variants are worth separate discussion. Only three germline mutations have been reported so far in ***AXIN2*** [23]. We report another putative pathogenic variant *AXIN2* p.R594W found in a patient with CRC at 45 years, however, we did not have information on existence of oligodontia in this carrier, a feature previously associated with AXIN2 germline mutations. All identified ***MUTYH*** variants were heterozygous and found in the cases with CRC before 50 without polyps. Prior reports have suggested an increased risk of CRC in *MUTYH* heterozygotes [24], so it appears that some *MUTYH* variants are more penetrant than others. While ***TGFBR2*** variant p.G169R was found in a young patient (MSS CRC at 31) from AC-I positive family, it is not conserved and predicted to be benign by PolyPhen2. While two ***POLD1*** and four ***POLE*** variants were found in exonuclease domain, *POLE* p.V2152M and p.R1077C in patients with young onset (<50 years) metachronous CRC were located outside of this domain. It is noteworthy that we found two mucinous adenocarcinomas of colon associated with ***POLE*** variants. ***MLH3*** involvement in hereditary CRC is still controversial [25], which may be explained by late age of onset of *MLH3* associated CRC. All six *MLH3* variants in our study were found in cases with MSS CRC diagnosed between 50 and 65 years, and three of the variants were found in cases that met AC-I criteria without age restriction. As expected, variants in *APC*, *POLE*, *POLD1* were found in FCCTX-like cases only; however, *MLH3* variants were also found in MSS CRC cases only, as well as variants in *CDH1*, *ALPK2*, and *SH2B3* candidate genes (Table 3). *CHEK2* and *ENG* variants were found in Lynch-like cases only. Several genes had variants in both FCCTX-like and Lynch-like cases including all MMR genes (*MLH1*, *MSH2*, *MSH6*, and *MSH3*), *HELQ*, *POLQ*, *LRIG1*, *PTCH1*, *LAMA2*, and *NOS1.* Some MMR variants found in FCCTX-like MSS cases may still be pathogenic, since MMR mutations have been reported in MSS CRC patients, especially those with *MSH6* mutations. However, in some cases better characterization of the cases is needed. For example *MSH6* p.R911^*^ mutation was found in cases 61 and 62 (Supplementary Table 2); while case 61 had MSI-High CRC, case 62 did not have MSI tested and misclassified as MSS ending up among Tier 3 cases.

***BAP1*** is a well-established tumor suppressor gene [26], and its downregulation is associated with decreased CRC survival [27]. A conserved variant *BAP1* p.R389C was found in a white male with CRC, squamous cell carcinoma, and basal cell carcinoma at age 66 years. This allele, detected by ExAC in only one European, is located in the same C-terminal hydrolase domain that harbors a mutation predisposing to melanoma, thyroid cancer, and mesothelioma [28–30]. Our finding raises a question regarding the role of *BAP1* germline mutations predisposing to squamous and basal cell carcinomas. It is noteworthy that two ***LAMA2*** variants p.R2578^*^ and p.I136M were detected in cases with both colon and prostate adenocarcinomas. ***CDH1*** is known to be associated with hereditary diffuse gastric cancer (HDGC) and other cancer types; however, none of the *CDH1* variant carriers had history of gastric cancer. ***CHEK2*** mutation p.R95^*^ has been described in breast cancer patients [31]; here, we report it in a patient with CRC at 52. ***ENG*** was found to cause Familial Juvenile Polyposis (FJP) with mutations in exons 11 and 12 reported in patients with late onset of CRC (60 and 68 years) and early age polyps (3 and 5 years) [32]. We found *ENG* exon 12 variant p.T550M in a case with polyps at 53 years and MSI-High CRC at 70 years. It is possible that *ENG* mutations are more common because early age polyps may remain undetected until the age colonoscopy surveillance starts. Our findings of variants in ***LRIG1***, ***PREX1***, ***NOS1***, and ***SH2B3*** further implicate these genes in hereditary CRC following the recent large GWAS [20]. However, additional segregation and functional studies are needed to confirm these results.

***BLM*** is a known tumor suppressor associated with higher CRC risk in heterozygotes, in addition to Bloom syndrome in homozygotes [33]. Recent study showed that heterozygote *BLM* mutations are associated with early onset CRC [34]. We found highly conserved *BLM* variants p.Y1044C and p.S897C (C-terminal helicase domain) in patients with metachronous CRC. Variants in the C-terminal helicase domain were found in both ***BLM*** and ***POLQ***. These findings suggest that C-terminal helicase domain mutations in *POLQ* and *BLM* may be involved in predisposition to hereditary CRC. It is noteworthy that identified *HELQ* variants were found outside of the helicase domain in carriers of candidate mutations in other genes.

DNA pooling has been previously used for genetic and genomic analysis including attempts to use DNA pooling for GWAS with mixed results, because pipetting errors interfered with estimated allele frequency. In NGS analysis pooling is frequently used as multiplexing with barcoded DNA samples pooled together to reduce the cost of sequencing. Pooling of non-barcoded samples is the further step to make sequencing even less expensive. It has been demonstrated that rare variants can be effectively identified in large populations using pooled NGS [35, 36]. Several guidelines and optimization algorithms for the analysis of rare variants in pooled NGS samples have been reported [37–39]. Previously, in our analysis of pooled exome sequencing, we showed that high depth of sequencing is important for identification of rare variants [40]. Therefore, our strategy is based on pooling DNA samples prior to preparation of the sequencing libraries followed by high depth sequencing and genotype validation, which makes our strategy more accurate and cost-efficient. A key factor in our strategy is that pooled sequencing is ideal for detection of very rare variants where the mere presence of the variant, and not its allele frequency, is important.

There are a number of limitations of the pooled sequencing strategy for analysis of very rare variants. Possibility that variants/mutations in genes not tested in this study account for the CRC in some of these cases cannot be excluded. For example, other candidate CRC genes including *RPS20* [41], *SEMA4A* [42] and *NTHL1* [14] have been published recently. Use of a specific percent of minor allele reads representing one heterozygote in a pool may lead to insufficient sensitivity of the analysis and some valid variants may be excluded. On the other hand, we used relatively relaxed frequency criteria (from 10% to 50% in a pool of three DNAs) to increase sensitivity and still >98% of the variants chosen for Sanger sequencing were validated. In addition, a number of genes, such as *EPCAM* and *GREM1*, have been reported to have copy number variations (CNVs) that have not been investigated in this study. The identified variants were predicted to be deleterious by at least one commonly used in silico variant effect prediction tool, however, it has been shown that in silico tools and their algorithms for missense variant effect prediction are only 65-80% accurate when examining known disease causing missense variants, therefore, further studies are needed before assigning pathogenicity to the missense variants identified in this study.

In conclusion, we performed a large targeted sequencing study using a DNA pooling strategy on 1046 CRC-affected cases selected for a positive family history of CRC and inclusive of both MSS and MSI-High subgroups of CRC. Our variant filtering criteria identified rare, predicted pathogenic variants in 106 cases representing 10% of all the cases tested. The cases with MMR gene variants comprised almost a quarter of the identified carriers, with other prominent genes identified in the established CRC genes group (MLH3 and POLE) and the candidate CRC genes group (NOS1, SH2B3, LAMA2 and MSH3) requiring further validation studies at both the gene and variant level. The DNA pooling NGS strategy applied in this study for identifying rare variants in hereditary CRC was a cost-effective approach for this large case-control targeted sequencing study and could be applied to other cancer types or complex diseases with a hereditary component, and may further facilitate studies aimed at identifying rare genetic risk factors in populations that are underrepresented in resequencing projects, such as Middle East or Slavic ethnicities. The results from our study support the concept that familial CRC is highly heterogeneous with regards to underlying genetic etiology with additional high-risk genes yet to be identified. Additional, large case-control studies supported by studies on functional effect and variant segregation are needed to generate the evidence needed to translate gene and rare variant discovery into improvements in clinical practice and actionability.

## MATERIALS AND METHODS

### Study population

The selection of CRC cases for this study was primarily based on CRC-affected individuals with a family history of CRC such as those who would be referred to Family Cancer Clinic and/or for genetic testing for hereditary CRC syndrome including individuals with both MSS and microsatellite unstable (MSI-H) tumors in order to provide findings of broad clinical relevance. We selected 1,046 familial CRC cases and 1,006 unrelated healthy controls from the Colon Cancer Family Registry Cohort (CCFR) [43]. Germline mutation testing for MMR gene mutations had been performed previously using different methods, including denaturing HPLC (dHPLC) screening prior to Sanger sequencing and MLPA during Phase II testing (2001-2006) while Phase III testing (2007-2012) involved Sanger sequencing and MLPA only; *MUTYH* gene testing involved genotyping and Sanger sequencing [43–45]. CRC cases were selected from six prioritized groups based on family structure of affected relatives, age at CRC diagnosis and tumor MMR status (Table 1). Therefore, the sample included FCCTX-like cases (MSS CRC patients from Tiers 1, 3, 4, and 5) and Lynch-like cases (MSI-H CRC patients from Tiers 2 and 6): Tier 1 included CRC patients with MSS tumors fulfilling Amsterdam criteria I (AC-I), also known as Familial Colorectal Cancer Type X [11], Tier 2 included AC-I or AC-II CRC patients with MSI-High tumors and no known MMR gene mutation or methylation of the *MLH1* gene promoter (Lynch-like), Tier 3 included CRC patients with MSS tumors from AC-I positive families without fulfilling the criteria of age (CRC <50 years), Tier 4 included AC-II CRC patients with MSS tumors, Tier 5 included CRC patients with MSS tumors from families with a proband <60 years old at CRC diagnosis and had ≥1 FDR or SDR with CRC, Tier 6 included CRC patients with an MSI-High tumor and no known MMR gene mutation or methylation of the *MLH1* gene promoter (Lynch-like) from families where proband had ≥1 FDR or SDR with CRC (although not fulfilling the AC-I or AC-II criteria). Controls were spouses or unrelated healthy individuals without cancer and selected to be older than cases at time of study (mean age 61 years) to account for variable CRC penetrance. Male to female ratio was 1:1 in cases and 1:1.5 in controls.

### Targeted sequencing and bioinformatics analysis

**Figure 2:**
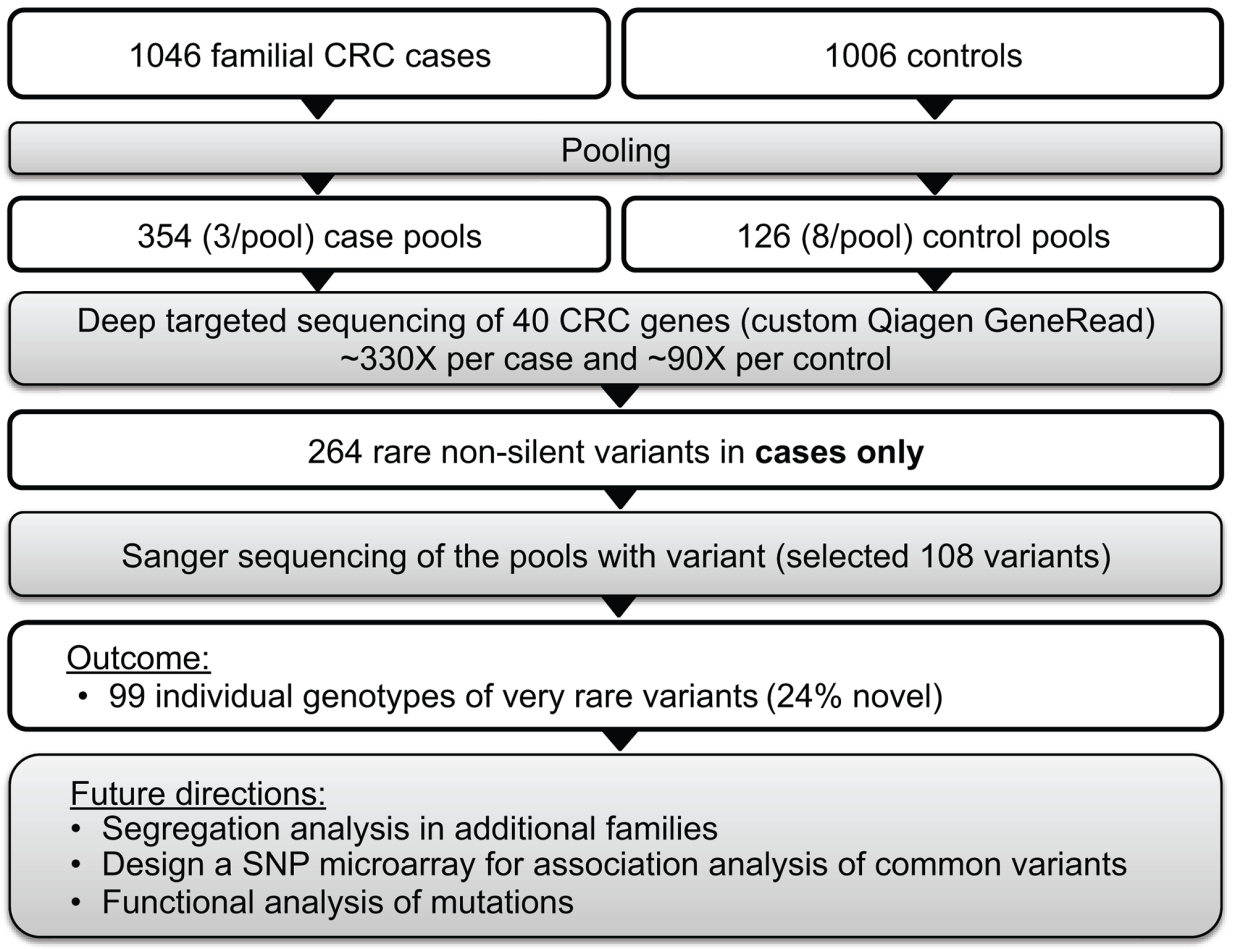
Targeted sequencing of pooled samples for identification of rare variants of large effect

We performed targeted sequencing of pooled DNA samples as a cost-efficient way to screen a large case-control sample set. Our strategy included four steps: 1) targeted sequencing of pooled cases and controls, 2) identification of case-only variants, 3) Sanger sequencing of case-only variants to identify individual genotypes and validate targeted sequencing (Figure 2). Blood-derived DNA from 1,046 cases and 1,006 controls were pooled into 480 pools in equimolar proportions (∼3 cases/pool and ∼8 controls/pool) for targeted sequencing of 40 established and candidate CRC genes (Table 2). Each sample was sequenced once. Genes were selected based on the following criteria: 1) established hereditary CRC genes (n=17), or 2) novel candidate CRC susceptibility genes (n=23) identified from the literature and those identified from whole exome sequencing study of familial CRC cases lead by the first author (Raskin et al., unpublished data). Custom libraries for each DNA pool were prepared using Qiagen GeneRead DNAseq Customized Targeted Panel covering exons and exon/intron boundaries and sequenced on the Illumina HiSeq 2500 to a mean depth of >1000X per case pool (>330X per case in each pool) and >700X per control pool (∼90X per control in each pool) at Vanderbilt Technologies for Advanced Genomics (VANTAGE). See Supplementary Methods and Supplementary Table 3 for more details.

Analysis of the raw sequencing data was performed at Vanderbilt Technologies for Advanced Genomics Analysis and Research Design (VANGARD) including a multi-stage quality control protocol developed previously [46, 47]. No quality concerns were observed. Alignments were performed using BWA against human genome reference hg19. We marked duplicates using Picard, then performed local realignment and local recalibration using the Genome Analysis Toolkit (GATK). Single nucleotide variants (SNVs) and indels were inferred using GATK’s Unified Genotyper. Results were further filtered based on GATK’s best practice. Annotations of SNV and indel were performed using ANNOVAR. Additional annotations were obtained through wANNOVAR [48], and Oncotator [49]. Variant reference databases including 1000 Genomes and ExAC were used as a source of variant allele frequency in addition to the controls tested. We selected all variants that had ≥2% reads with alternative allele to exclude false positives. Fractions of the alternative allele were calculated per pool for further analysis (alternative allele reads/total reads). A minor allele percentage in a case pool reads between 10% and 50% and ≥50 reads were used as a threshold to select variants. Likely deleterious variants were defined as variants (nonsense, frame-shift, splice-site variants) likely to result in protein truncation or disrupt a consensus splice site (i.e. +/- 1, 2) and non-synonymous variants predicted to be pathogenic according to MutationTaster, PolyPhen-2, and SIFT *in silico* tools from ANNOVAR. Rare likely deleterious variants identified in only the CRC cases from targeted sequencing were tested by Sanger sequencing to exclude a false positive variant and to confirm which CRC case in the DNA pool was the carrier (Sanger sequencing primers available on request).

American College of Medical Genetics and Genomics (ACMG) recommended an updated standards and guidelines for interpretation of sequence variants as benign or pathogenic based on the following types of data: population, computational and predictive, functional, segregation, *de novo* status, and presence in other databases [19]. ACMG guidelines were used for interpretation of the clinical significance of the variants.

## SUPPLEMENTARY MATERIALS FIGURES AND TABLES









## Abbreviations

CRC: colorectal cancer
NGS: next-generation sequencing
MSI: microsatellite instable
MSS: microsatellite stable
MMR: mismatch repair
FCCTX: familial colorectal cancer type X
CCFR: Colon Cancer Family Registry
ACMG: American College of Medical Genetics
