# Supplementary material for: Targeted sequencing of established and candidate colorectal cancer genes in the Colon Cancer Family Registry Cohort
Source: Oncotarget. 2017 Jun 21;8(55):93450–63. doi: 10.18632/oncotarget.18596 (PMC5706810; doi:10.18632/oncotarget.18596)
Supplement: Supplementary file 1 [file oncotarget-08-93450-s001.pdf]

Targeted sequencing of established and candidate colorectal cancer genes in the Colon Cancer Family Registry Cohort

SUPPLEMENTARY MATERIALS

Targeted sequencing library construction and quality statistics

DNA pools were treated as individual DNA samples for NGS library construction using custom GeneRead DNAseq Targeted Panel from Qiagen. The system employs overlapping primer sets across the exons to maximize target coverage (multiplex PCR-enabled target enrichment of genomic regions of interest). Overlapping primer sets are divided into an appropriate number of pools to maximize specificity. Following amplification and purification, enriched regions from each sample are pooled together, yielding one library preparation for each sample (description from Qiagen). The custom primer mix was prepared to cover exons and exon/intron boundaries of the selected 40 genes. DNA samples were combined with primer mix and PCR reagent followed by PCR amplification. PCR products were purified and used for NGS library construction and sequencing using Illumina HiSeq 2500. Sequencing results were aligned against the reference human genome HG-19. Mean target coverage was 176 reads across all 480 samples. Mean number of the total reads was 262,042 with 98% of reads being on target. On average over 50% of the targets were covered at least 100X. The detailed targeted NGS quality statistics is presented in Supplementary Table 3.

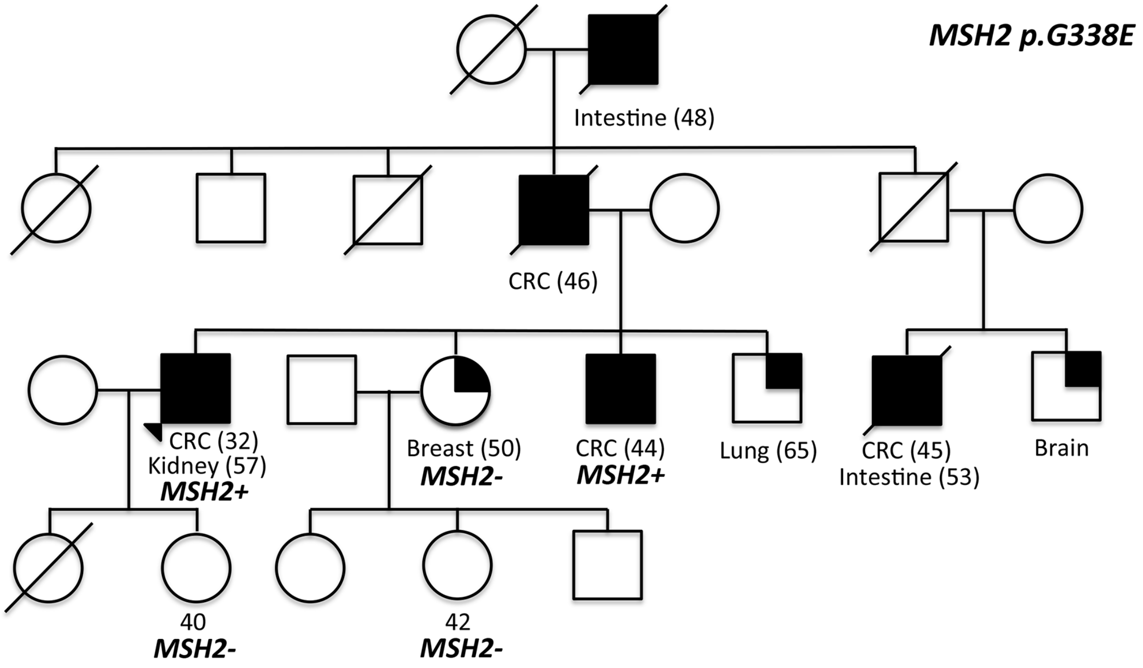

Supplementary Figure 1: Segregation analysis of *MSH2* p.G338E variant.

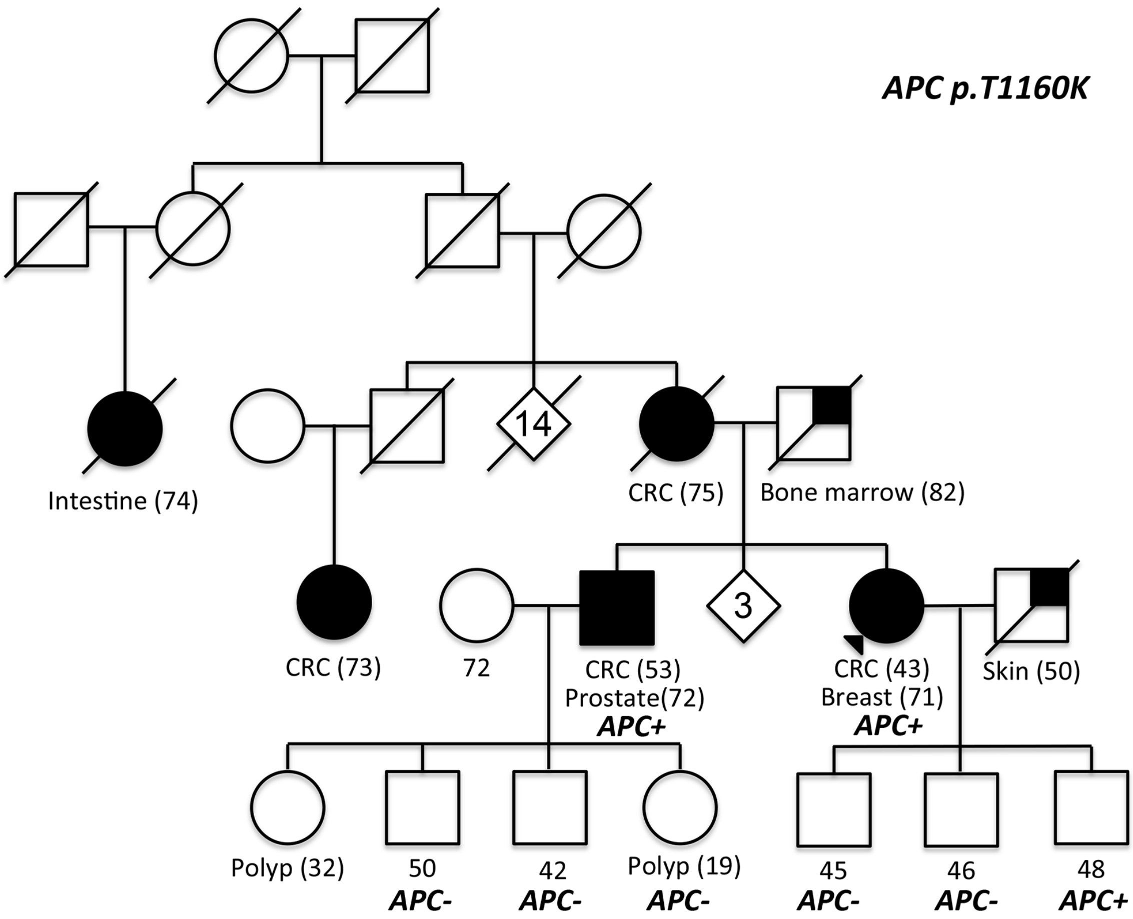

Supplementary Figure 2: Segregation analysis of *APC* p.T1160K variant.

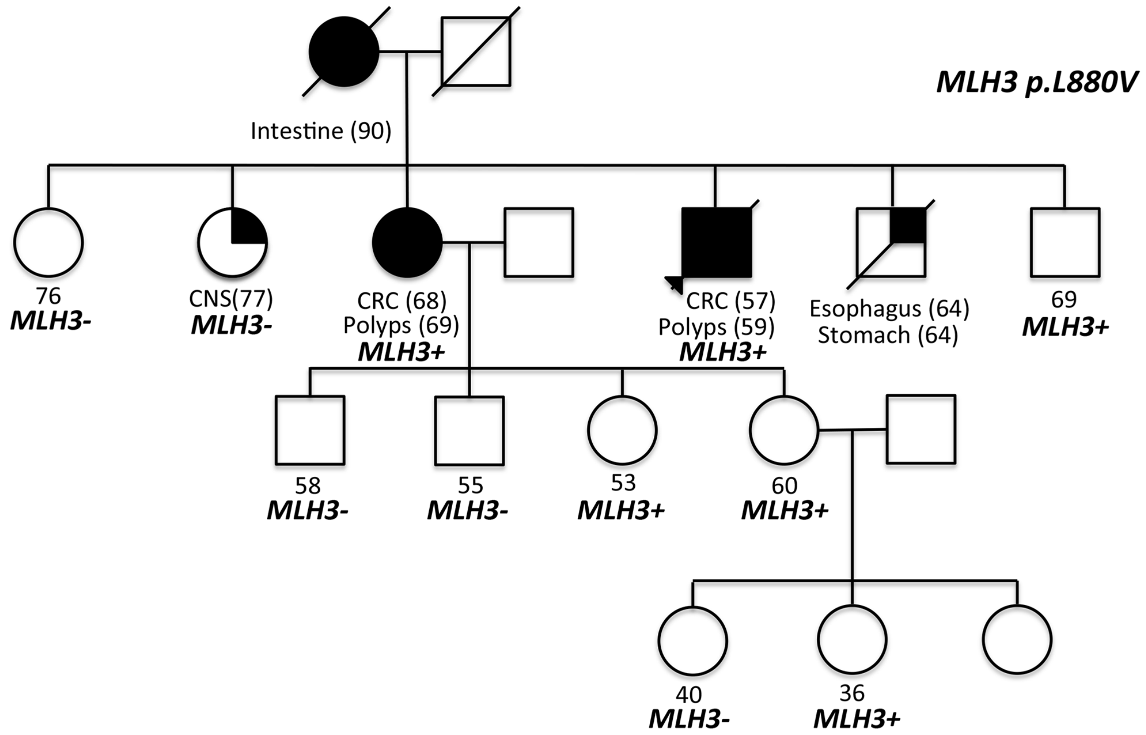

Supplementary Figure 3: Segregation analysis of *MLH3* p.L880V variant.

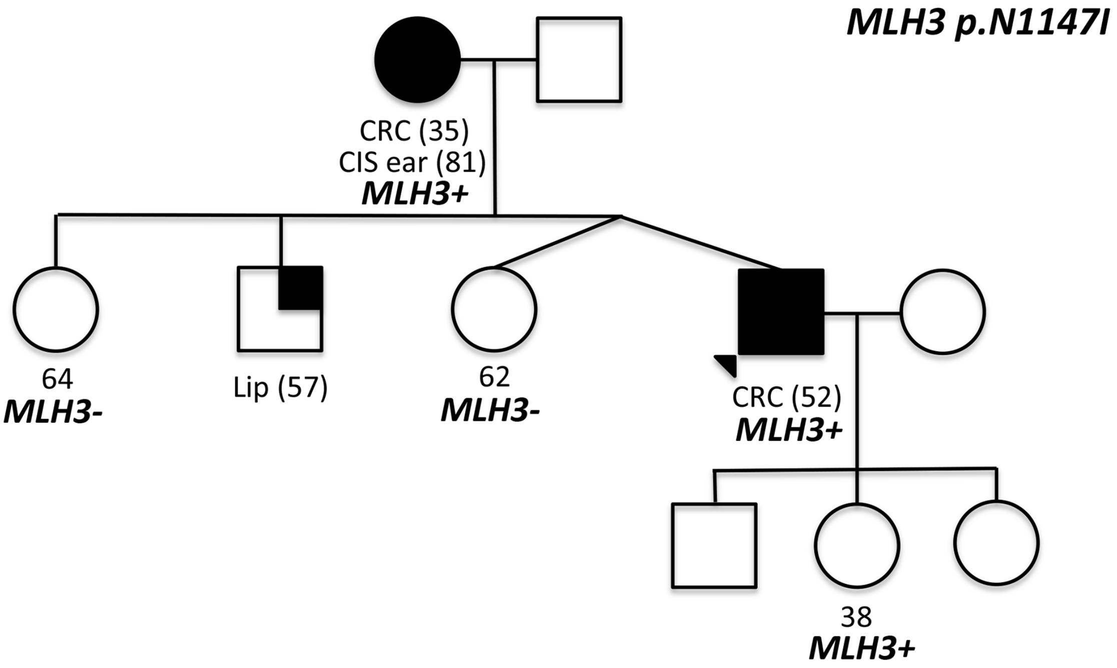

Supplementary Figure 4: Segregation analysis of *MLH3* p.N1147I variant.

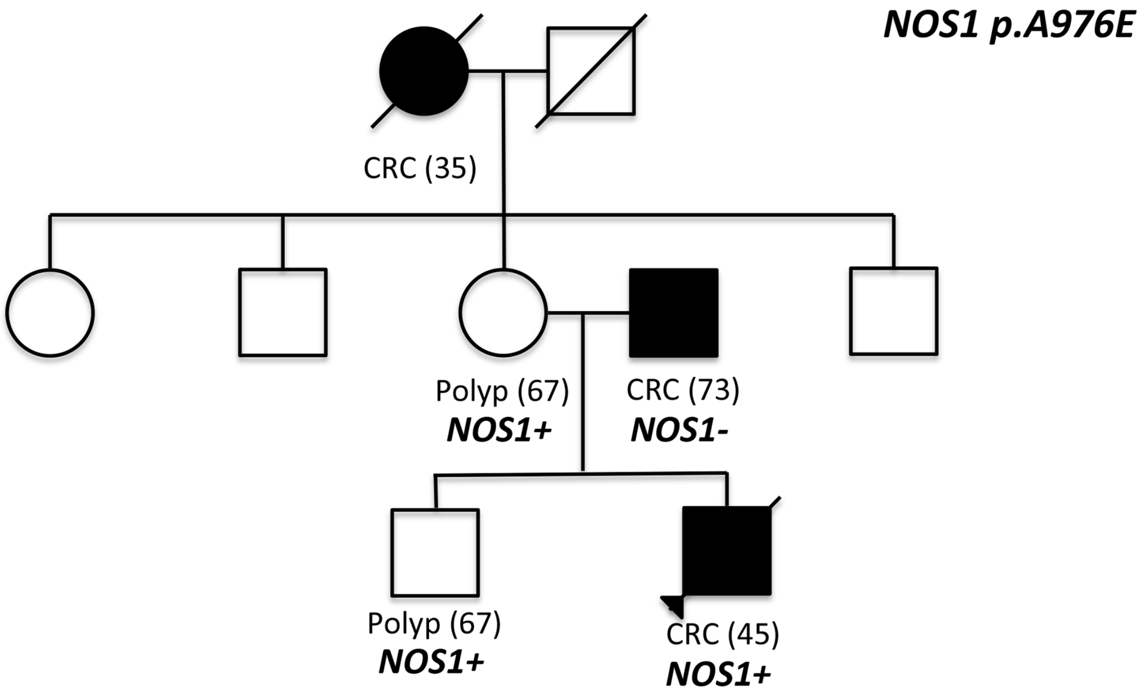

Supplementary Figure 5: Segregation analysis of *NOS1* p.A976E variant.

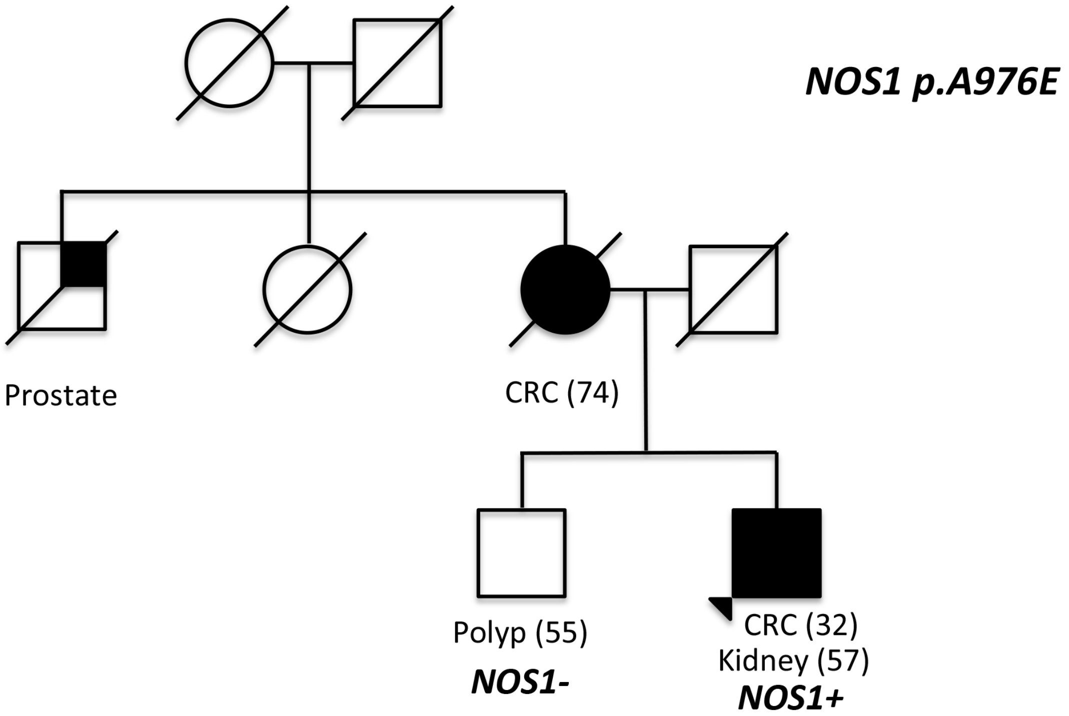

Supplementary Figure 6: Segregation analysis of *NOS1* p.A976E variant.

Supplementary Table 1: All identified rare variants.

See Supplementary File 1

Supplementary Table 2: Variants validated by Sanger sequencing with individual genotypes.

See Supplementary File 2

Supplementary Table 3: Alignment summary of targeted sequencing.

See Supplementary File 3
